# The complete mitochondrial genome of the coral *Goniopora planulata*

**DOI:** 10.1080/23802359.2022.2080020

**Published:** 2022-06-02

**Authors:** Wenjing Huang, Mingyao Meng, Yanping Zhang, Cheng Shen, Li Liu

**Affiliations:** a Fisheries College, Guangdong Ocean University, Zhanjiang, China; b Xuwen National Coral Reef Nature Reserve, Zhanjiang, China; c Southern Marine Science and Engineering Guangdong Laboratory, Zhanjiang, China

**Keywords:** *Goniopora planulata*, mitogenome, phylogenetic analysis

## Abstract

Widely distributed in marine neritic areas, the coral *Goniopora planulata* (Ehrenberg, 1834) is a vulnerable species on the International Union for Conservation of Nature (IUCN) Red List. This study sequenced the circular mitogenome of *G. planulata*. The full genome length was 18,766 bp, with an overall base composition of 25.6% for A, 13.7% for C, 23.5% for G, and 37.2% for T; GC content was low at 37.2%. 20 genes were identified, including 13 protein-coding genes (PCGs), 2 ribosomal RNA genes, and 5 transfer RNA genes. Phylogenetic analysis of the complete *G. planulata* mitogenome can aid in understanding evolutionary relationships within Scleractinia.

*Goniopora planulata*, commonly found in the Indo-Pacific and South China Seas, is a colorful coral that forms small compacted columns or mounds (Veron and Staffordsmith 2000). Four *Goniopora* mitochondrial genomes have been reported: *G. columna* (Lin et al. 2011), *G. sitokesi* (Chen et al. 2019), *G. djiboutiensis* (Tong et al. 2019), and *G. lobata* (Peng et al. 2020).

Climate change and anthropogenic disturbance have seriously damaged *G. planulata* habitats, leading to population decline. The coral has thus been classified as vulnerable on the Red List of Threatened Species by the IUCN. In this study, we sequenced the *G. planulata* mitogenome and analyzed its evolutionary relationship with other Scleractinia species. Obtaining this data is important for coral conservation.

Samples used in this study were collected from Xuwen County, Zhanjiang City, Guangdong Province (109°5012″E, 20°1036″N). They were transported to and stored at the Key Laboratory of Aquaculture in the South China Sea for Aquatic Economic Animal of Guangdong Higher Education Institutes, Guangdong Ocean University (Zhanjiang, Guangdong), under voucher BPJK_XW2005302 (Wenjing Huang, huangwenjing_1996@163.com). All procedures strictly followed guidelines in the ‘Laboratory Animal Treatment and Usage Guide of Guangdong Ocean University.’ The Animal Ethics Committee of Guangdong Ocean University approved the study protocol.

Next-generation whole-genome sequencing was performed using the Illumina platform, with the *G. lobata* mitogenome (GenBank accession number MN795054) as reference. Reads were assembled in GetOrganelle version 1.7.5 (Jin et al. 2019), and transfer RNA sequences were confirmed using tRNAscan-SE (Schattner et al. 2005). The assembled genome was annotated in MITOS2 (Bernt et al. 2012).

The complete circular *G. planulata* mitogenome (GenBank accession number OL372279) was 18,766 bp in length. Overall base composition was 25.6% A, 13.7% C, 23.5% G, and 37.2% T, with low GC content (37.2%). The mitogenome consisted of 20 genes (13 protein-coding genes [PCGs], 2 ribosomal RNA genes, and 5 transfer RNA genes). Nine PCGs had a typical initiation codon (ATG), whereas two used GTG (*nad3* and *nad4L*), one used ACC (*nad5*), and one used ATA (*nad6*). The stop codon for all PCGs was either TAA or TAG.

To validate the phylogenetic position of *G. planulata*, comparisons were made with other Scleractinia mitochondrial sequences. We used MAFFT v7 with TIM3 + F + R3 model for sequence alignment and used MEGA-X to construct a Maximum-likelihood tree with 1000 bootstrap replicates (Kumar et al. 2018). The analysis included complete mitogenomes of 20 species from 13 different families in Scleractinia. Soft coral *Briareum asbestinum* was set as an outgroup (Figure 1).

The result shows that *G. planulata* has a close relationship with *G. columna*, *G. djiboutiensis*, and *G. sitokesi*, while *G. lobata* has a remoter relationship with *G. planulata*.

In conclusion, this study provided valuable insight into the phylogenetic relationship between *G. planulata* and other species of Scleractinia. Our results should benefit the conservation of *G. planulata* and offer a basis for future research on this vulnerable coral species.[Fig F0001]

## Authors’ contributions

Concept and design: Wenjing Huang, Yanping Zhang, Li Liu; Provision of study materials: Cheng Shen; Data analysis and curation: Wenjing Huang, Mingyao Meng, Yanping Zhang; Drafting of the manuscript: Wenjing Huang, Mingyao Meng; Revision of the manuscript: all authors; Final approval of the version to be published: all authors; Agreement to be accountable for all aspects of the work: all authors.

## Data Availability

Genome sequence data are publicly available in GenBank under accession number OL372279 (https://www.ncbi.nlm.nih.gov/nuccore/OL372279.1/). The associated BioProject, SRA, and Bio-Sample numbers are PRJNA792901, SRR17371972, and SAMN24471554, respectively. All databases are located at NCBI (https://www.ncbi.nlm.nih.gov/). Molecular phylogeny of *Goniopora planulata* and other species in Scleractinia based on complete mitogenomes downloaded from GenBank. The phylogenetic tree was constructed using the Maximum-likelihood method with 1000 bootstraps. Letters and numbers preceding species names are the GenBank accession numbers.
